# Environmental Tobacco Smoke and Educational Self-Regulation and Achievement in First Grade High School Students

**DOI:** 10.25122/jml-2020-0020

**Published:** 2020

**Authors:** Mina Rashidi, Asghar Mohammadpoorasl, Mohammad Hasan Sahebihagh

**Affiliations:** 1. Student Research Committee, Department of Community Health Nursing, Tabriz University of Medical Sciences, Tabriz, Iran; 2. Health and Environment Research Center, Tabriz University of Medical Sciences, Tabriz, Iran; 3. Tabriz Health Services Management Research Center, Department of Community Health Nursing, Tabriz University of Medical Sciences, Tabriz, Iran.

**Keywords:** Environmental tobacco smoke, passive smoking, educational self-regulation, educational achievement

## Abstract

Environmental tobacco smoke, containing many toxic gases, suggests inevitable contact of humans with the damaging factors of cigarettes. On average, approximately 40% of children, 35% of women and 32% of men worldwide are exposed to environmental tobacco smoke. This study aims at investigating the relationship between environmental tobacco smoke in adolescents and their educational self-regulation and achievement. In this study, 770 students aged between 13 and 15 were selected and studied using the multistage sampling method. The tools used in this study consisted of four questionnaires, demographic characteristics, environmental tobacco smoke, educational self-regulation, and educational achievement. The validity and reliability of tools have been approved, and the data were analyzed using SPSS v22. The results indicated a significant inverse relationship between environmental tobacco smoke and students’ educational self-regulation and achievement (p-value > 0.001). Given the relationship between exposure to environmental tobacco smoke and educational self-regulation and achievement, it is essential to keep children away from tobacco smoke. Family health and education policy-makers are recommended to design and operate fundamental schemes in order to deal with environmental tobacco smoke.

## Introduction

In 2000, a US general surgeon issued 11 health pieces of advice to raise global awareness of important public health issues, 7 of which were related to tobacco smoke exposure [1]. Environmental tobacco smoke (ETS) contains many toxic chemical gases, including carbon monoxide, hydrogen cyanide, butane, ammonia, benzene, and toluene. The toxic metals they contain include lead, chromium, arsenic, and cadmium, indicating the inevitable contact of humans with the damaging factors of cigarettes [2]. Exposure to ETS increases the levels of blood lead of the youth, which will potentially lead to tragic cognitive consequences [3]. It is predicted that carbon monoxide in secondhand smoking (SHS) can bind to hemoglobin to form carboxyhemoglobin in the blood, which may cause reduced oxygenation to the brain and affect mental functioning [4]. On average, approximately 40 percent of children, 35 percent of women, and 33 percent of men worldwide are exposed to ETS [5]. The consequences of children’s exposure, in particular to SHS, are a major potential health-care challenge [6]. In our society, the number of women smokers is low. However, a lot of male smokers seem to have exposed women and children to cigarette smoke indirectly [7]. Cars are also a place where children are exposed to toxic constituents of cigarettes, and car space constraint exacerbates the issue[8]. In addition, the levels of particles of cars can be well above the WHO-recommended level [9]. Parental smoking in the first 7 years of life after childbirth was significantly associated with symptoms of attention deficit and hyperactivity disorders and contradictory behaviors [10, 11]. Similarly, the findings of Rockinger et al. suggest that children aged 10 who had been exposed to cigarette smoke after birth showed hyperactivity and inattention behaviors almost twice as much as children who lived with non-smoking parents [12]. In a study on US children under 12 years of age about parents’ report of smoking at home after childbirth and its association with diagnosed neurobehavioral disorders (attention deficit and hyperactivity disorders, learning disability, and behavioral disorder), the findings suggested that children exposed to SHS at home showed a 50 percent higher chance of developing two or more childhood psychological disorders than non-exposed children [13]. Recent studies showed that adolescents’ cognitive disorders were not associated with exposure to prenatal tobacco smoke early in life; however, it was associated with SHS exposure during adolescence [4]. The findings of a study on maternal cigarette smoking during pregnancy and cognitive function in adolescence showed that maternal cigarette smoking did not affect their children’s cognitive abilities during adolescence [14]. Still, exposure to ETS during adolescence affected their cognitive abilities [15]. Research shows that passive smoking causes problems similar to active smoking [16]. In fact, the hazards of ETS are approximately equal to those of active smoking, and children who are in their early years of growth are more vulnerable than adults [17]. Even exposure to ETS can cause cochlear damage and hearing loss in children [3].

There are several potential mechanisms, including the formation of carboxyhemoglobin in the blood, oxidative stress, inflammation, and atherosclerotic processes related to other toxic compounds in SHS such as hydrogen cyanide, arsenic, cadmium, lead, ammonia and vinyl chloride[18]. The findings show that exposure to ETS is a nervous system suppressor and has a negative effect on sensory processing. In addition, exposure to cigarette smoke in humans is associated with an increased risk of cognitive and attention impairment as well as sensory processing, and it also causes psychiatric disorders such as ADHD [19]. Despite efforts in housing reconstruction and environmental remediation as well as reduced cigarette smoking and SHS exposure in the United States, cigarette smoke remains an essential source of lead exposure in vulnerable populations and the population in general [3]. Given the complications of ETS and its effect on mental abilities and attention deficit and hyperactivity in children, we decided to study the rate of exposure to ETS in students and its relation to educational achievement and self-regulation.

## Material and Methods

This research is a descriptive study that was conducted in the spring of 2018 on the first-year male and female students of six public schools in Malekan (a city at the north-west of Iran). 770 male and female students aged 13 to 15 were studied. According to the sample size, the number of schools and the students in each school sampling was done in a random cluster manner. Each cluster consisted of one school, in which all schools were selected, and classes were randomly selected from each school, and all students were selected from each class. Given that each school had an average of 8 classes and the number of students per class was 30, five classes were randomly selected from each school to reach the desired sample size. The inclusion criteria included first-year high school students as well as those who had at least one smoker in the family, and exclusion criteria included students who had smoked either during their study or during the past three months.

Four questionnaires were used in this study:

1. Demographic characteristics: Demographic characteristics consisted of gender, age, number of close friends, father’s education, mother’s education, presently living with which family member, smoking status in the family, the person’s status of smoking hookah, and the status of the person’s smoking and their friends.
2. Exposure to ETS: In this study, nine questions were asked by the researcher to measure the amount of ETS. This questionnaire was designed according to two standard questions from the World Youth Tobacco Survey [20]. These questions show the status of exposure to ETS at home, outdoors, in cars in the past 24 hours, in the past week, and during the lifetime.
3. Educational self-regulation questionnaire: A self-regulation questionnaire containing 30 items was used to measure the variable of educational self-regulation. This questionnaire was designed and developed through the study of some items of external questionnaires and interviews with some high school students in Ahvaz, and finally, exploratory factor analysis. This questionnaire consists of 30 questions and six factors called “memory strategy” (5 questions) with a score range of 5-25, goal-setting (3 questions) with a score range of 3-15, self-assessment (6 questions) with a score range of 6-30, help-seeking (6 questions) with a score range of 6-30, accountability (4 questions) with a score range of 4-20, and organization (6 questions) with a score range of 6-30. The questionnaire was scored on a Likert scale, with scores of 5, 4, 3, 2, and 1 for “strongly agree”, “agree”, “undecided”, “disagree”, “strongly disagree”. The total score range was 30-150.
4. Educational achievement: In this study, students’ end-of-semester grade point average, as well as science and mathematics scores of the same semester, was used to measure their educational achievement. The validity and reliability of questionnaires were measured by content validity and test-re-test (r=0.7).

To conduct the research, the researcher went to the intended schools after obtaining the necessary permits from the Directorate-General of Education in East Azerbaijan Province and the Department of Education of Malekan. Having justified the goals and coordinated with the schools and after the sampling classes were specified, the researcher went to the selected class. The aims of the study were explained to students and teachers, and they were assured that the information in the questionnaires would remain confidential. The data collected through questionnaires and analyzed with appropriate statistical techniques. At the level of descriptive statistics, the frequency percentage, mean, and standard deviation were used, and at the level of analytical statistics, t-test and ANOVA were used. The analysis was performed using SPSS v22. This scheme was ethically approved by the Ethics Committee based in the Chancellor of Research and Technology of Tabriz University of Medical Sciences (IR.TBZMED.REC.1396.1126).

## Results

Out of the 770 students, 71 were excluded due to tobacco smoking, and 52 were excluded due to unacceptable reporting of the duration of exposure to ETS. Therefore, 647 students were assessed. 49.6% and 50.4% of participants were boys and girls, respectively. The majority of participants had low socio-economic status (50.8%). 94.5% of the participants lived with both parents, and 46.2% of the participants lived at home with a smoker, and most of them did not have any smoking friends (93%). In this study, 50.8% of children were exposed to ETS, and 49.2% of them were not. Participants’ exposure to ETS was 30 minutes (0.5 hours) per day, 72 minutes per week, and 406 hours in the lifetime. 54 and 46 percent of participants reported less than 15 minutes and more than 15 minutes of exposure per week to ETS, respectively. The majority of children exposed to ETS were exposed to their smoking fathers’ smoke in the home (59.3%), and only two children were exposed to their smoking mother’s environmental smoke. The number of smoking sisters was zero in this study, and only 9.8% of the students were exposed to the cigarette smoke of other smokers. In this study, exposure to ETS did not have a significant relationship with the socio-economic status, gender, and having a smoking friend. However, it had a significant relationship with the status of living with parents and the presence of a smoker in the home (P-value<0/001) (Table 1).

**Table 1:** Demographic status of participants and the mean exposure to ETS (based on hours).

Variable		Abundance		Hours of exposure to environmental smoke		P-value
		Number	Percentage	Average	SD	
Sex	Male	310	49.6	431.9	766.95	0.966
	Female	311	50.4	380.35	761.85	
Socio-economic level	Low	311	50.8	456.05	798.35	0.343
	Middle	154	25.5	373.6	718.12	
	High	145	23.7	357.03	758.36	
Living with parents	Yes	579	94.5	415.23	775.73	<0.017
	No	33	5.5	265.23	440.73	
Smoker in the family	Yes	277	46.2	801.33	955.48	<0.001
	No	344	53.8	87.75	305.41	
Having a friend Smoker	Yes	42	7.0	561.50	845.96	0.484
	No	575	93.0	387.88	750.40	

The findings of this study showed that the highest exposure to ETS occurs at home (35.4%), and students are exposed to ETS in the house for an average of 12 minutes a day (Table 2). Overall, 47.7% of the participants were exposed to ETS in the house, outdoors, and in the car for an average of 30 minutes daily. The findings of this study showed that there was a significant inverse relationship between exposure to ETS and students’ educational self-regulation, science and math scores, and the grade point average (P>0.001). In addition, there was a direct and significant relationship between educational self-regulation and science and math scores and the grade point average (Table 3).

**Table 2:** The frequency of the location of exposure to ETS in participants and the average exposure hours.

**Location of Exposure**	**Frequency of exposure to environmental smoke**		**Average hours of exposure to environmental smoke**					
			**In the last 24 hours**		**In the week**		**In a lifetime**	
	**Number** N = 647% *	Percentage*	**Average**	**Standard deviation**	**Average**	**Standard deviation**	**Average**	**Standard deviation**
**Home**	226	35.4	0.23	0.66	1.01	4	474.3	2211.88
**Outside the house**	165	26.3	0.14	0.42	0.27	1.03	74.8	242.52
**Car**	179	28.4	0.14	0.59	0.26	1.06	62.83	214.87
**Total**	323**	50.8	0.47	0.99	1.19	2.69	406.05	764.22

* shows the column for exposure in each location and, generally, 50.8% of participants had exposure at least in one of the three locations.

** One can have simultaneous exposure in all the three locations. Therefore, the total exposure will not be equal to the algebraic sum of the three locations.

**Table 3:** The relationship between ETS and participants’ educational self-regulation and achievement (Malekan, 1397).

	**The amount of environmental smoke**	**Academic self-regulation**	**Half year grade point average**	**Math score**
**Academic self-regulation**	r =-0.370 P-value <0/001			
**Half year grade point average**	r =-0.335 P-value <0/001	r =0.505 P-value <0/001		
**Math score**	r =-0.376 P-value <0/001	r =0.509 P-value <0/001	r =0.818 P-value <0/001	
**Science score**	r =-0.341 P-value <0/001	r =0.453 P-value <0/001	r =0.804 P-value <0/001	r =0.790 P-value <0/001

## Discussion

This study aims at determining the amount of exposure to ETS in first-year high school students of the city of Malekan and its relationship with educational self-regulation and achievement. According to the present study, the amount of students’ exposure to ETS was 50.8%. A study in Scotland showed that 40.2% of participants were exposed to ETS [21]. The exposure to ETS in Iran as a developing country seems to be higher than that in Scotland as a developed country.

In the present study, most people were exposed to ETS less than 15 minutes per week. A study conducted in Jiangsu Province, China, in 2014 showed that 48% of participants were exposed to SHS for at least 15 minutes per week, and most people had less than 15 minutes of exposure to ETS[22]. The results of this study are consistent with those of the present study. However, given that the prevalence of smoking cigarette in China (more than 300 million or 22 percent of the population) [23] is higher than Iran (approximately 11 million or 12% of the population) [24], the level of exposure to ETS is equal in these two countries. This may indicate that cigarette smokers in Iran are less likely to care about the people around them than those in China.

In the present study, the highest exposure to ETS occurred at home. The results of a survey conducted in China showed that approximately 56% of Chinese youths were exposed to SHS in public spaces, and 44% were exposed at home [23]. Given that the outdoor environment was divided into two groups, namely inside the car and public space, in the present study, the total exposure in these two environments was 54.7, which is consistent with the above survey. In a study conducted in Sri Lanka in 2018, the findings suggested that exposure to SHS in this country was higher in public places than at home [25].

According to the results of this study, the average exposure to ETS in children living with a smoker in the house was higher than in children who did not live with any smokers at home, and the only smoker in the family was often the father and only two smoking mothers were reported. A study conducted in Mongolia showed that 49.2% of families lived with at least one smoker at home, most of whom were the fathers of the family, and only 3 smoking mothers were reported [26]. In addition, a survey conducted at 12 schools in Dhaka, Bangladesh, suggested that 43% of children lived with at least one smoker in the home, who was the father in 99% of cases [27]. Aycicek concluded in his study that the most important source of child exposure to cigarette smoke is parental smoking [8]. Even if parents smoke outside the house, children can still experience high levels of exposure to ETS [28]. The findings of all these studies are consistent with the results of the present study, indicating that the home and parents, especially the father of the family, are key sources of SHS in Bangladesh, Mongolia, Iran, and probably elsewhere in the world.

In the current study, 35.4% of children reported being exposed to ETS at home. In their research, Saito et al. reported that 7.6% of children in the United States, 12.7% in the United Kingdom, and 14.4% in Japan were exposed to SHS in the house [29], which shows that in Iran, as a developing country, exposure to ETS at home is higher than that of the countries listed as developed countries. ETS not only affects the health of those exposed to SHS but also contaminates the home of smokers up to 6 months after smoking the last cigarette and remains on surfaces and dust [30].

According to the World Health Organization, having separate rooms for smoking as well as having an air conditioning system is not effective in preventing exposure to SHS [27]. As maintained by a study conducted in Bandar Abbas in 2011 on people over the age of 15, cars were the most common places of exposure to ETS [31], which was inconsistent with the results of the present study, and this may have cultural roots. ETS exposure varies widely across countries and has a robust socio-economic slope, and in more impoverished socio-economic conditions, children have the highest rate of exposure to ETS [32]. In our study, exposure to environmental cigarette smoke decreased with the enhancement of socio-economic status as well. However, this relationship was not statistically significant. This insignificant relationship might have been due to the small sample size and high dispersion of exposure to ETS, as well as high standard deviation.

The findings of this study showed an inverse significant relationship between the mean exposure to ETS and the mean of students’ educational self-regulation and achievement. In a study by Sai Yin Ho et al., the findings showed that SHS exposure was associated with more reduced educational performance in non-smoking adolescents [20], which was consistent with the results of the present study.

The results of this study also showed that with increasing self-regulation, educational achievement increases in many aspects, including students’ science and math scores as well as their grade point average. According to a theory by Zimmerman et al., self-regulating students are far superior to other students whose learning is not self-regulated [33]. Given that exposure to ETS disrupts students’ self-regulation and educational performance and reduces their chances of success, it can be stated that smokers at home deprive students of higher education set forth in the Universal Declaration of Human Rights (Article 26) [20].

## Conclusions

In summary, as indicated by the results of the present study, exposure to ETS decreases students’ self-regulation and educational failure. According to the above findings, exposure to ETS can be considered as a risk factor for adolescents, and corrective measures can be taken to reduce or eliminate exposure. One limitation of our study was the students’ self-report of exposure to ETS. Given the possibilities of the present study, this research has been limited to asking individuals questions and obtaining a record of them. However, to compensate for this limitation, it has been suggested that researchers use urine or saliva cotinine in order to calculate the exposure to ETS.

However, the findings of the present study were different from those of Ahmadizadeh et al. [31]. Given that smoking is influenced by cultural patterns, it is suggested to research this in other parts of Iran in order to understand the issue of ETS better. Overall, the study of ETS has received little attention from researchers, and its association with other quantitative indices of great health has not received much attention from researchers. The present study suggests that researchers across the world should quantitatively consider ETS, and its relationship with health indices such as prevalence and mortality in infants and children should be investigated as well. In this study, given that exposure to ETS in Iran as a developing country was higher than that in the United Kingdom, Japan, the United States, and Scotland as developed countries, it is suggested that exposure to ETS in developing and developed countries should be studied comparatively. Smoking cigarettes is a risk factor whose effects are often long-term. Therefore, it is suggested to investigate the relationship between ETS and variables such as quality of life, life satisfaction, happiness, and general health.

Although we found a significant inverse relationship between exposure to environmental smoke and educational self-regulation and achievement in this study, it needs to be repeated in different populations for conducting systematic review researches and obtaining robust results.

## Acknowledgments

We would like to express our gratitude to the Department of Research and Technology of Tabriz University of Medical Sciences for funding this research as well as the Department of Education of Malekan for their contribution to the present study. We would also thank all the students who sincerely participated in this study.

## Conflict of Interest

The authors confirm that there are no conflicts of interest.
